# Complete Genome Sequences of the Prototype Isolates of Genotypes 2, 3, and 4 of Murray Valley Encephalitis Virus

**DOI:** 10.1128/genomeA.00581-14

**Published:** 2014-06-19

**Authors:** David T. Williams, Sinéad M. Diviney, Karli J. Corscadden, Beng Hooi Chua, John S. Mackenzie

**Affiliations:** a Australian Biosecurity CRC for Emerging Infectious Disease, Curtin University, Perth, Western Australia, Australia; b School of Biomedical Sciences, Faculty of Health Sciences, Curtin University, Perth, Western Australia, Australia

## Abstract

Murray Valley encephalitis virus (MVEV) (*Flaviviridae* family, *Flavivirus* genus), a mosquito-borne pathogen of humans and horses, is endemic to the Australasian region. We report here the complete genomes of the prototype strains of MVEV genotypes 2, 3, and 4.

## GENOME ANNOUNCEMENT

Murray Valley encephalitis virus (MVEV) (*Flaviviridae* family, *Flavivirus* genus), a member of the Japanese encephalitis serocomplex, is a significant arboviral pathogen capable of causing severe neurological infections in humans and horses (1, 2). MVEV exists in transmission cycles involving *Culex* species mosquitoes and waterbirds (3). It is enzootic in northern Australia and Papua New Guinea (PNG) and is occasionally epizootic in southeastern Australia (4). Four genotypes (G1 to G4) of MVEV have been recognized (5–8). G1 is the dominant genotype circulating in mainland Australia. Recent isolates from PNG also belong to G1 (6). G2 is a minority genotype and consists of mosquito isolates from the Kimberley region of Western Australia (6). Single strains of MVEV from PNG comprise G3 and G4; additional strains belonging to these lineages have not been identified. Only one full-length genome of MVEV has been reported, that of the prototype strain MVE-1-51, which belongs to G1 (9). Here, we report the full-length genomic sequences of the prototype strains of each of the three other genotypes: OR156 (G2), NG156 (G3), and MK6684 (G4). OR156 was originally isolated from *Culex annulirostris* mosquitoes trapped at Kununurra in the Kimberley region of Western Australia in 1973 (10). The PNG strain NG156 (also referred to as MVE-1-56) is a human brain isolate from a fatal case of MVE in Port Moresby in 1956 (11), while MK6684 is an isolate from mixed culicine mosquitoes trapped at the Japanaut village on the Sepik River in 1966 (I.D. Marshall, unpublished data).

Viral RNA extracts from infected Vero cell culture supernatants were reverse transcribed using MVEV-specific primers or random hexamers (GeneWorks, Australia). PCR amplification was performed with overlapping primer pairs designed using the genome sequence of MVE-1-51 (Genbank accession no. AF161266) and partial sequences of OR156, NG156, and MK6684 (Genbank accession no. EF015074 to EF015076 and L48974 to L48976) as references. Sequencing was performed using purified PCR amplicons (Australian Genome Research Facility [AGRF], Australia). The 5′ and 3′ termini were sequenced using a modified RACE method (12).

The genomes of MK6684 and MVE-1-51 are 11,014 nucletodies (nt) long, whereas that of NG156 is 11,012 nt in length, and that of OR156 is the shortest at 10,953 nt. Deletions in the 3′ untranslated region (UTR) previously reported for MK6684 and OR156 (13) were confirmed, including a 63-nt deletion in the highly variable region downstream of the open reading frame (ORF) stop codon of the OR156 genome, as well as additional insertions and deletions in the 3′ UTR. In the 5′ UTR, a single uracil insertion was found at position 53 of the MK6684 and NG156 genomes, and a double uracil insertion was found at this site for OR156. The size and position of each of the viral genes contained in these strains were identical compared to each other and to those in MVE-1-51. Nucleotide sequence analysis revealed the highest levels of identity between MK6684 and NG156 (94%). OR156 shared the lowest levels of nt identity with other full-length genomes (88.1 to 88.4%). We envisage that the availability of these genome sequences will allow for a better understanding of MVEV molecular ecology.

### Nucleotide sequence accession numbers.

Each of the MVEV genomes are deposited in GenBank, with accession no. KF751869 (for MK6684), KF751870 (for NG156), and KF751871 (for OR156).
